# Rifampicin and Its Derivative Rifampicin Quinone Reduce Microglial Inflammatory Responses and Neurodegeneration Induced In Vitro by α-Synuclein Fibrillary Aggregates

**DOI:** 10.3390/cells8080776

**Published:** 2019-07-25

**Authors:** Leonardo Acuña, Sabah Hamadat, Natalia S. Corbalán, Florencia González-Lizárraga, Mauricio dos-Santos-Pereira, Jérémy Rocca, Julia Sepúlveda Díaz, Elaine Del-Bel, Dulce Papy-García, Rosana N. Chehín, Patrick P. Michel, Rita Raisman-Vozari

**Affiliations:** 1 Institut du Cerveau et de la Moelle épinière (ICM), Inserm U 1127, CNRS UMR 7225, Sorbonne Université, F-75013 Paris, France; 2 Instituto de Patología Experimental, CONICET/Universidad Nacional de Salta (UNSa), Salta A4408FVY, Argentina; 3 Laboratoire Croissance, Régénération, Réparation et Régénération Tissulaires (CRRET)/ EAC CNRS 7149, Université Paris Est Créteil, Université Paris Est, 94010 Créteil, France; 4 Instituto de Medicina Molecular y Celular Aplicada (IMMCA) CONICET/UNT and SIPROSA, Tucumán T4000ILI, Argentina; 5 Faculdade de Medicina de Ribeirão Preto, Universidade de São Paulo, São Paulo 04023-062, Brazil

**Keywords:** aggregation, α-synuclein, microglia, neuroinflammation, Parkinson’s disease, cytokines, neuronal survival

## Abstract

Aggregated forms of the synaptic protein α-synuclein (αS) have been proposed to operate as a molecular trigger for microglial inflammatory processes and neurodegeneration in Parkinson´s disease. Here, we used brain microglial cell cultures activated by fibrillary forms of recombinant human αS to assess the anti-inflammatory and neuroprotective activities of the antibiotic rifampicin (Rif) and its autoxidation product rifampicin quinone (RifQ). Pretreatments with Rif and RifQ reduced the secretion of prototypical inflammatory cytokines (TNF-α, IL-6) and the burst of oxidative stress in microglial cells activated with αS fibrillary aggregates. Note, however, that RifQ was constantly more efficacious than its parent compound in reducing microglial activation. We also established that the suppressive effects of Rif and RifQ on cytokine release was probably due to inhibition of both PI3K- and non-PI3K-dependent signaling events. The control of oxidative stress appeared, however, essentially dependent on PI3K inhibition. Of interest, we also showed that RifQ was more efficient than Rif in protecting neuronal cells from toxic factors secreted by microglia activated by αS fibrils. Overall, data with RifQ are promising enough to justify further studies to confirm the potential of this compound as an anti-parkinsionian drug.

## 1. Introduction

Parkinson’s disease (PD) is a neurodegenerative disease clinically characterized in part by motor symptoms resulting from the loss of dopaminergic neurons in the substantia nigra pars compacta. In addition to neuronal demise, inflammatory processes and intraneuronal protein accumulation are characteristic histopathological hallmarks of PD [1]. Proteinaceous inclusions localized in the neuronal perikarya (Lewy bodies) and in neuronal processes (Lewy neurites) are primarily composed of α-synuclein (αS) [2]. The aggregation of αS in the central nervous system (CNS) is a pathological process of fundamental importance to the development and progression of PD [3] and other synucleinopathies. The αS aggregates are thought to favor neurodegeneration either by directly damaging neurons or by inducing the production of inflammatory mediators (e.g., TNF-α and reactive oxygen species (ROS)) that are neurotoxic [4,5]. These aggregates are generally surrounded by activated microglia and immune cells in the CNS [6,7], which suggests that they may operate as a trigger for neuroinflammatory processes and neurodegeneration to promote PD progression [8].

Besides their bactericidal activity, some antibiotic molecules also possess anti-inflammatory, anti-aggregant, or antioxidant properties, which may be of potential interest for PD treatment [9,10,11,12,13]. Such is the case, for instance, of rifampicin (Rif), a macrocyclic bactericidal antibiotic that is widely used in the treatment of infectious diseases, especially those caused by *Mycobacterium,* including tuberculosis and leprosy. Rif has been reported, for example, to attenuate cerebrospinal fluid (CSF) concentrations of inflammation markers as well as neuronal damage in children with bacterial meningitis [14]. Rif was also the first antibiotic reported to have therapeutic effects in chronic neurodegenerative conditions. In particular, leprosy patients subjected to a chronic treatment with Rif displayed a significantly decreased prevalence of dementia [15,16]. To date, a number of studies have reported anti-inflammatory effects of Rif in paradigms that are pertinent to neurological diseases [17,18,19,20]. Still, mechanisms through which Rif could reduce neuroinflammation processes observed in neurodegenerative diseases remain to be clarified.

**Figure 1:**
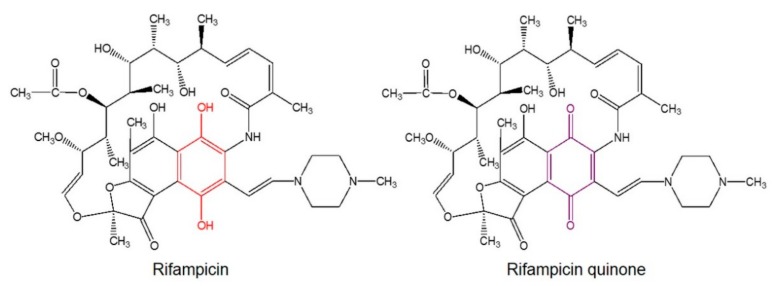
Rif and RifQ chemical structures. The naphthyl core structure of Rif becomes a naphtoquinone in RifQ. This signifies that the para-diphenol (red) of the naphthyl core is converted into a para-quinone (violet).

Interestingly, when Rif is dissolved in aqueous solution, an oxidation process occurs spontaneously, leading to the formation of different oxidized species, including rifampicin quinone (RifQ) [21]. This molecule differs from Rif in that the naphthyl core structure is converted into a naphtoquinone (Figure 1). This confers to the molecule distinctive biochemical properties.

Although many studies have demonstrated the anti-inflammatory effects of Rif, few experimental data have been obtained so far with RifQ. Rif was described as a potential immunosuppressive agent in rats, but these effects were observed only with stored (i.e., not freshly prepared) solutions of the antibiotic. Therefore, the anti-inflammatory effects of Rif were ascribed to an oxidation product of Rif, RifQ [22]. Likewise, other studies indicated that an oxidation product of Rif was preventing the fibrillation of αS and promoting the disaggregation of already formed fibrils [21].

In the present study, we wanted to compare the potential of Rif and RifQ to modulate microglial inflammation induced by αS fibrils (αS_f_). Therefore, using microglial cells in culture [23], we first evaluated the effects of αS_f_ on microglial activation and found that Rif, and to a larger extent RifQ, were able to resolve inflammatory processes in part by inhibiting phosphatidylinositol-3-kinase (PI3K)/protein kinase B (AKT). Besides, we found that RifQ protected cortical neurons exposed to toxic factors secreted by αS_f_-activated microglia.

## 2. Materials and Methods

Leibovitz’s L-15 medium, Dulbecco’s modified Eagle’s medium (DMEM), Trypsin-EDTA 0.05%, and penicillin/streptomycin cocktail were all purchased from Invitrogen Life Technologies (Saint Aubin, France). Fetal calf serum (FCS) was obtained from Biowest LLC (Eurobio, Les Ulis, France). Poly(ethyleneimine) (PEI) (average molar mass 750,000, P3143), lipopolysaccharide (LPS; *E. coli* strain O26:B6; L8274), Rif R0700000, RifQ R0800000, LY-294,002 hydrochloride (LY), 2′(3′)-O-(4-Benzoylbenzoyl)adenosine 5′-triphosphate triethylammonium salt (bz-ATP), Hoechst 33342, the Cell Counting Kit-8, the rabbit Anti-Glyceraldehyde-3-phosphate dehydrogenase antibody (anti-GAPDH), and the Superoxide anion assay kit were all purchased from Sigma Aldrich (L’Isle d’Abeau Chesnes, France). The interleukin (IL)-6 and tumor necrosis factor (TNF)-α enzyme-linked immunosorbent assay (ELISA) kits, the M-PER™ Mammalian Protein Extraction Reagent, Pierce™, the Limulus Amebocyte Lysate (LAL) Chromogenic Endotoxin Quantitation Kit, the Pierce BCA Protein Assay Kit, and the Pre-stained Protein Ladder were obtained from ThermoFisher Scientific (Saint-Herblain, France). The rabbit anti-ionized calcium binding adaptor molecule-1 (IBA-1) antibody (#019-19741) was from Wako (Neuss, Germany). The rabbit phospho-AKT (Ser473) antibody (#9271) and rabbit AKT antibody (#9272) were purchased from Cell Signaling Technology (Saint-Quentin Yvelines, France). The monoclonal IgG isotype 2a anti-mTLR2-IgG antibody (α-TLR2) and the TLR2 agonist Pam3CSK4 were obtained from InvivoGen (Toulouse, France), and 2-(Phenylthio)-*N*-[[tetrahydro-4-(4-phenyl-1-piperazinyl)-2*H*-pyran-4-yl]methyl-3-pyridinecarboxamide JNJ 47965567 (JNJ) was purchased from Tocris Biosciences (Bristol, UK).

### 2.1. Preparation of α-synuclein

Expression and purification of recombinant human αS was performed as previously described [24] and endotoxins potentially present were removed using a high capacity endotoxin removal resin (ThermoFisher) following the manufacturer´s instructions. Then, protein samples were filtered, centrifuged for 30 min at 12,000× *g*, and residual endotoxins were quantified using the Limulus Amebocyte Lysate assay. Monomeric αS stock solutions containing less than 0.1 endotoxin unit (EU)/mg protein were prepared in 20 mM 4-(2-Hydroxyethyl)piperazine-1-ethanesulfonic acid (HEPES), 150 mM NaCl, pH 7.4. The protein concentration was determined by measurement of absorbance at 280 nm using an extinction coefficient ε_275_ of 5600 cm^−1^ M^−1^. Protein aggregation was performed using αS solutions (1 mg/ml) diluted in 20 mM HEPES, 150 mM NaCl, pH 7.4. Samples were incubated in a Thermomixer C (Eppendorf) at 37 °C under constant orbital agitation (600 revs/min) to obtain fibrillary aggregates. The aggregates were then sonicated for 2 min in an ultrasonic bath and kept at −20 °C until further use.

### 2.2. Transmission Electron Microscopy

Samples (50 μl) of a 1 mg/ml αS solution were adsorbed onto glow-discharged 200 mesh formvar/carbon coated copper grids (Electron Microscopy Sciences, Hatfield, PA) and stained with uranyLess (Electron Microscopy Sciences). Excess liquid was removed and grids were allowed to air dry. Samples were viewed and imaged using a Hitachi 7700 transmission electron microscope (Hitachi, Tokyo, Japan).

### 2.3. Cell Culture Protocols

#### 2.3.1. Microglial Cell Isolation

Animals were housed, handled, and cared for according to the recommendations of the European Union Council Directives (2010/63/EU). The experimental procedures were authorized by the ethical committee for animal experiments Charles Darwin n°5.

Pure microglial cell cultures were obtained as previously described using a technique that relies on the preferential adhesion of microglia to the polycation polyethyleneimine (PEI) [23]. Briefly, the brains of postnatal day 1 C57BL/6J mouse pups (Janvier LABS, Le Genest St Isles, France) were harvested, and the meninges stripped away, after which brain tissue was mechanically dissociated by repeated pipetting. After two rounds of trituration, the supernatant containing the dissociated cells was centrifuged at 1000 rpm for 5 min at 4 °C. The resulting pellet was triturated and resuspended in DMEM supplemented with 10% heat-inactivated FCS and 1% penicillin/streptomycin solution (defined as complete medium). Then, a cell suspension obtained by trituration of 2 mouse brains was plated in each PEI-coated T-75 culture flask (Sigma-Aldrich) containing complete medium. The cultures were washed once with complete medium after 2 days in vitro and microglial cells were then maintained at 37 °C in a humidified atmosphere with 5% CO_2_ without any other culture medium change until completion of isolation. The isolation was generally obtained 14–18 days after plating under these conditions. The average yield was approximately 4–5.10^6^ cells/T-75 culture flask with this protocol.

#### 2.3.2. Microglial Cell Stimulation and Treatments

After isolation, microglial cells were harvested by trypsinization and seeded onto uncoated 48-well plates (Nunc) at a density of 10^5^ living cells per well, unless otherwise specified. The αS species (70 μg/ml), bz-ATP (500 μM), and Pam3CSK4 (1 μg/ml) were applied the day after plating for 24 h. Unless otherwise specified, Rif and RifQ were used at 100 µM, a concentration found to elicit optimal anti-inflammatory effects in preliminary experiments. The concentrations used for LY (2.5 μM), α-TLR2 (2.5 μM), and JNJ (20 μM) were based on previous work [25]. Treatments with Rif, RifQ, LY, α-TLR2, and JNJ were initiated 3 h before applying the inflammogens to the cultures.

#### 2.3.3. Primary Cortical Neuron Cultures

Primary cultures of cortical neurons from embryonic day 16 C57BL/6J mouse fetuses were prepared according to the protocol employed by Fifre et al. (2006) [26]. Dissociated cells were seeded at a density of ~10^5^ cells/cm^2^ onto 48-well plastic plates that were precoated with 1.5 g/ml of poly-DL-ornithine. Cortical cells were cultured without serum using a chemically defined DMEM-F12 medium (Life Technologies) that was supplemented with salts, hormones, and proteins, as described previously [26]. Cultures were maintained at 37 °C in a humidified 5% CO_2_ atmosphere.

#### 2.3.4. Neurotoxicity Assays

After 6 days in vitro, the seeding medium of cortical neurons was replaced by a culture medium conditioned by microglial cultures that were exposed or not to treatments of interest. Then, after 48 h of incubation with microglial conditioned medium (CM), cell viability was monitored with the Cell Counting Kit-8 (CCK-8, Sigma-Aldrich) according to the manufacturer’s instructions. Briefly, CM was removed and the CCK-8 solution (15 µl of CCK-8 reagent in 150 µl culture medium) was added to each well for an incubation of 3 h at 37 °C in a humidified atmosphere with 5% CO_2_. After termination of the incubation, the supernatant from each well was transferred into a 96-well microplate, and the absorbance measured at 450 nm using a Tecan’s Infinite® 1000 spectrometer (Tecan Group, Männedorf, Switzerland).

#### 2.3.5. Microglial-Conditioned Media Preparation

Primary microglial cells were plated and maintained with the same chemically-defined medium as that used for cortical cultures. After 16 h, microglial cells were treated for 3 h with Rif or RifQ followed by a stimulation with αS_f_ for 24 h. After that, the supernatants were immediately transferred to cortical cultures for neurotoxicity assays. To verify the degree of activation of microglial cells in this setting, we measured the TNF-ɑ and IL-6 levels in the supernatants. Note that Rif and RifQ were used at 10 µM in CM experiments. This is because we found that these concentrations are sufficient to inhibit microglial cell responses elicited by αS_f_ in serum-free conditions.

### 2.4. Protein Detection by Immunofluorescence

After the termination of treatment, the cultures were fixed with 4% formaldehyde (12 min, 4 °C), washed with PBS, and incubated overnight with antibodies against IBA-1 (1:500 in PBS with 0.02% Triton X-100) to detect microglial cells. As secondary antibody, we used a goat anti-rabbit Alexa Fluor -555 (Invitrogen). When needed, nuclei of labelled cells were counterstained with Hoechst 33342 (10 µM). Phase contrast and fluorescent images were acquired using a Nikon TE 2000 inverted microscope (Nikon, Tokyo, Japan) equipped with an ORCA-ER digital camera and HCImage imaging software (Hamamatsu Corp., Bridgewater, NJ, USA).

### 2.5. Western Blot Analysis

The culture supernatants were removed, and microglial cells were washed with PBS and lysed using the M-PER™ Mammalian Protein Extraction Reagent. Protein contents of the samples were quantified using a bicinchoninic acid (BCA) Protein Assay kit. Equal amounts of protein were separated by electrophoresis on a 10% sodium dodecyl sulfate polyacrylamide gel electrophoresis (SDS-PAGE), followed by transfer to a nitrocellulose membrane. The membranes first blocked in PBS containing 50% Odyssey blocking buffer (blocking solution) (LI-COR® Bioscience), were then incubated overnight at 2–8 °C with primary antibodies (1:1000) against Glyceraldehyde-3-phosphate dehydrogenase, anti-ionized calcium binding adaptor molecule-1, phospho-AKT, or AKT diluted in the blocking solution. After incubation, the membranes were washed with a Tris-buffered saline Tween-20 solution and incubated with an adequate amount of Infrared Fluorescent (IR)Dye secondary antibody (LI-COR®). We used an Odyssey CLx near-infrared fluorescence imaging system (LI-COR®) for Western blot imaging and quantification. Immunofluorescent signals were normalized according to protein levels in control conditions (untreated cells).

### 2.6. NADPH Oxidase Activity

Reduced nicotinamide adenine dinucleotide phosphate (NADPH) oxidase activity was determined with the superoxide anion assay kit (Sigma Aldrich) by measuring chemiluminescence products formed through the oxidation of luminol by superoxide anions. Briefly, cells were seeded (1.5.10^5^ cells/well) in white opaque 96 wells microplates, pre-incubated 1 hour with Rif (100 µM), RifQ (100 µM), or LY (2.5 µM), and stimulated with αS_f_. Twenty-four hours later, the culture medium was removed and the cultures washed with a Hank’s balanced salt solution. Living cells were then incubated with 100 µl of medium assay provided by the assay kit. The enzymatic reaction was triggered by addition of luminol and the chemiluminescence signal was monitored at 37 °C using a microplate reader SpectraMax M4 (Molecular Devices, Sunnyvale, CA, USA), with one acquisition every 2 s for 300 min. 

### 2.7. Statistical Analysis

Data were analyzed by one-way ANOVA followed by the Tukey post-hoc test. All data are presented as mean ± SEM of at least 3 independent experiments, except when noted. Statistical analysis was performed with the Statistix 9.0 software.

## 3. Results

### 3.1. α-Synuclein Fibrils Induce Pro-Inflammatory Cytokine Release in Microglial Cell Cultures

**Figure 2:**
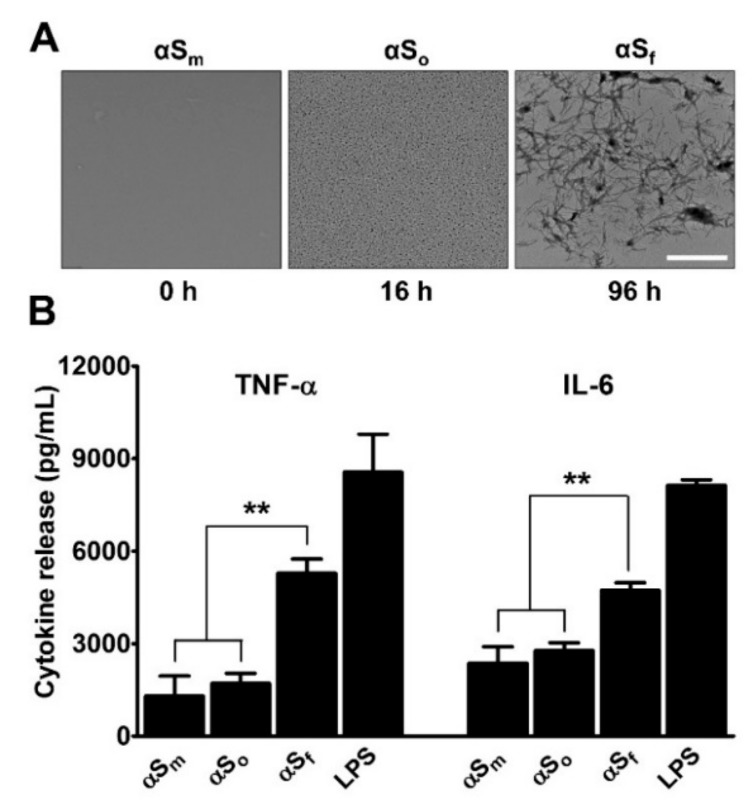
Impact of α-synuclein species on microglial cytokine release. (**A**) Transmission electron microscopy images of αS (70 µg/ml) samples agitated at 37 °C by orbital shaking and harvested after 16 h or 96 h. Images show the presence of oligomers (αS_o_) and fibrillary species (αS_f_), at 16 and 96 h, respectively. Scale bar: 1 µm. (**B**) Impact that samples of αS (70 µg/mL) harvested after 16 h (αS_o_) or 96 h (αS_f_) of orbital agitation and non-shaken samples (αS_m_) have on the release of tumor necrosis factor (TNF)-α and Interleukin (IL)-6 in microglial cell cultures. Cytokine release was near to zero in control cultures. Lipopolysaccharide (LPS) (10 ng/mL) was utilized as a positive control for stimulation. Data are means ± standard error of the mean (SEM) of three independent experiments performed in triplicate. Note: **p < 0.01 vs. αS_f_.

Recombinant monomeric αS (αS_m_) (70 µg/ml) was agitated by orbital shaking as previously described [10], and aggregation kinetics monitored by measurement of Thioflavin T (ThT) fluorescence [27] (not shown). The αS oligomers (αS_o_) and αS_f_ were harvested after 16 and 96 h of incubation at 37 °C, respectively, and structural states were confirmed by transmission electron microscopy (TEM) (Figure 2A).

Next, we measured the effect that a 24 h treatment with αS_m_, αS_o_, or αS_f_ had on the release of two pro-inflammatory cytokines TNF-α and IL-6 in PEI-isolated microglial cell cultures. We used lipopolysaccharide (10 ng/mL) as reference inflammogen [28]. Cytokines were quantified in the culture supernatants using enzyme-linked immunosorbent assay kits (Figure 2B). Importantly, the treatment of microglial cells with αS_f_ for 24 h strongly promoted the release of TNF-α and IL-6. Note that cytokine release was also increased but to a smaller extent by αS_m_ and αS_o_ (*p* < 0.01 vs. αS_m_ or αS_o_). Based on these results, we used αS_f_ as inflammogen in subsequent studies. Note that αS_f_ appeared somehow less effective than LPS (10 ng/mL) in stimulating cytokine release in this specific setting.

### 3.2. Rifampicin and Rifampicin Quinone Prevent Microglial Activation Induced by α-Synuclein Fibrils

**Figure 3:**
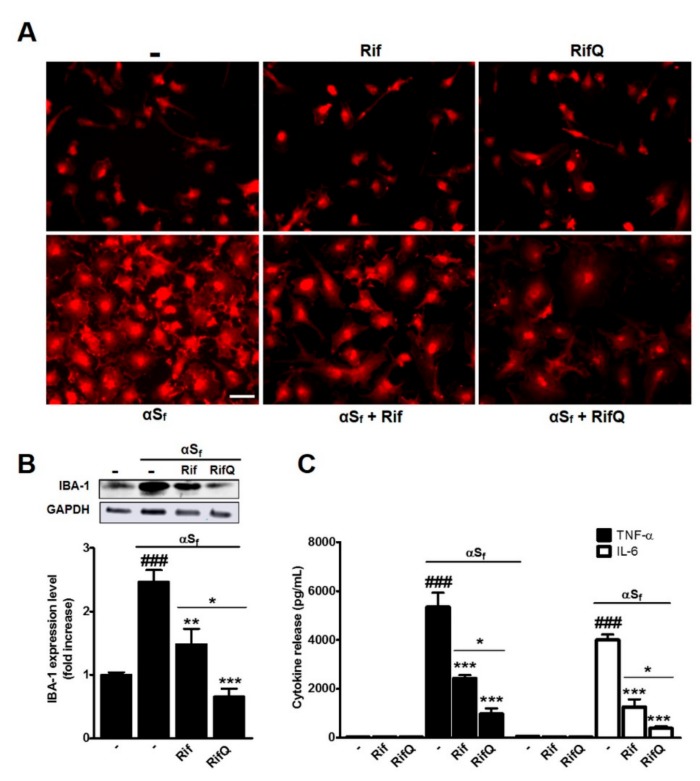
Inhibitory effects of Rif and RifQ on αS_f_-activated microglial cells. (**A**) Visualization of the effects of Rif and RifQ (100 µM) on anti-ionized calcium binding adaptor molecule-1 (IBA-1) (red) immunofluorescent signals in microglial cells stimulated or not with αS_f_. A treatment with Rif or RifQ reduced the increase in IBA-1 expression observed in cultures exposed to αSf. Rif and RifQ had and no effect on basal IBA-1 expression levels. Scale bar: 20 µm. (**B**) Western blot analysis showing the extent of the inhibitory effects of Rif and RifQ on IBA-1 expression in microglial cells exposed to αS_f_. A representative blot is included (top), and relative intensity levels of IBA-1 are shown (bottom). (**C**) Effect of 100 µM Rif and RifQ on the amounts of TNF-α (filled bars) and IL-6 (open bars) released by microglial cells stimulated by αS_f_. Values are shown as means ± SEM of four independent experiments performed in triplicate. Note: ^###^*p* < 0.001 vs. controls, *p < 0.05 Rif vs. RifQ. **p < 0.01 and ***p < 0.001 vs. αS_f_.

To assess the anti-inflammatory effects of Rif and RifQ, we first quantified expression levels of IBA-1, a macrophage and microglia activation marker that is classically upregulated during neuroinflammatory processes [29,30]. In line with previous observations [25], αS_f_ strongly increased IBA-1 expression, as monitored by fluorescence immunostaining (Figure 3A) and western blot imaging (2.46-fold vs. controls) (Figure 3B), which is indicative of a global inflammatory state in these cultures. The treatment of microglial cells with 100µM Rif or RifQ restrained IBA-1 induction by αS_f_, as evaluated by immunofluorescence staining (Figure 3A). Note that neither Rif nor RifQ had a significant effect on basal IBA-1 expression. Western blotting quantification revealed that RifQ (*p* < 0.01 vs. αS_f_) was much more effective than Rif (*p* < 0.001 vs. αS_f_) in counteracting the effects of αS_f_ on IBA-1 expression (*p* < 0.05) (Figure 3B).

To better assess the extent of the anti-inflammatory effects of Rif and RifQ toward αS_f_-activated microglial cells, we also measured TNF-α and IL-6 levels in the supernatants of cultures receiving the same treatments as before. Consistent with previous results, αS_f_ caused a robust increase in the release of both cytokines in microglial cultures. Treatments with either 100 µM Rif or RifQ significantly reduced the levels of secreted cytokines (*p* < 0.001 vs. αS_f_) (Figure 3C). Similar to what we observed with IBA-1, the inhibitory effect of RifQ on TNF-α and IL-6 release was proportionally greater than that of Rif (*p* < 0.05).

### 3.3. Rifampicin and Rifampicin Quinone Prevent TLR2- and P2X7-Dependent Microglial Activation 

**Figure 4:**
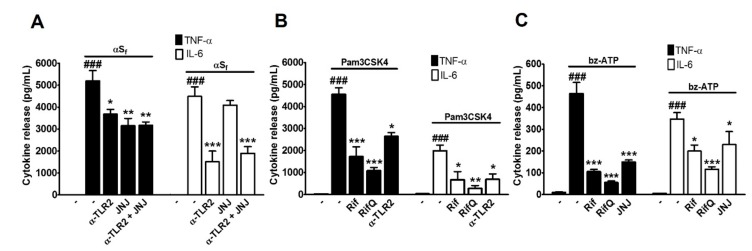
Inhibitory effects of Rif and RifQ on microglial cells activated by either αS_f_ or agonists of putative receptors for αS_f_. (**A**) Modulation of the effects of αS_f_ by α-TLR2 (2.5 μg/ml), an antagonistic antibody against TLR2 and by JNJ 47965567 (JNJ) (20 μM), a synthetic antagonist of P2X7 receptors. (**B**) Modulation of the effects of Pam3CSK (1 µg/ml) on TNF-α and IL-6 release in microglial cell cultures treated with Rif, RifQ (both at 100 µM), or the TLR2 receptor antagonist (α-TLR2; 2.5 µg/ml). (**C**) Impact of a treatment with bz-ATP (500 µM), a specific agonist for P2X7 receptors, on TNF-α and IL-6 release in microglial cell cultures treated with Rif, RifQ (both at 100 µM), or the P2X7 receptor antagonist JNJ (20 µM). Values are shown as means ± SEM of four independent experiments performed in triplicate. Note: ^###^*p* < 0.001 vs. control, *p < 0.05, **p < 0.01, and ***p < 0.001 vs. corresponding inflammogen, i.e., αS_f_, Pam3CSK4, or bz-ATP.

We previously reported the presence of αS aggregates close to the plasma membranes of microglial cells, which suggests that aggregated species had the capacity to activate plasma membrane receptors. In fact, we found that TLR2 and P2X7 receptors were involved in the release of glutamate induced by αS_f_ in microglial cells [25]. Consistent with these observations, we were able to demonstrate, here, that the effects of αS_f_ on cytokine release were partly antagonized by α-TLR2 (2.5 μg/ml), an antagonistic antibody for TLR2, and by JNJ (20 μM), a synthetic antagonist for P2X7 receptors (Figure 4A). The inhibitory effects of α-TLR2 were observed on TNF-α (*p* < 0.05 vs. αS_f_) or IL-6 (*p* < 0.001 vs. αS_f_) release induction by αS_f_. JNJ was, however, mostly effective on TNF-α release induction (*p* < 0.01 vs. αS_f_). Note that the combination of the two antagonistic treatments did not further improve cytokine release inhibition.

These observations led us to test the potential of Rif and RifQ in a situation where microglial cell activation was due to the stimulation of TLR2 or P2X7 receptors. For that microglial cell cultures were challenged with Pam3CSK4, a synthetic TLR2 agonist [31] or 2′(3′)-O-(4-Benzoylbenzoyl)adenosine 5′-triphosphate triethylammonium (bz-ATP), a synthetic ATP analogue, which is an agonist of P2X7 receptors [32].

Pam3CSK4 (1 μg/ml) and Bz-ATP (500 μM) led to robust and moderate stimulation of cytokine release, respectively (*p* < 0.001 vs. controls) (Figure 4B,C). Rif and RifQ (100 µM) significantly reduced TNF-α levels in the supernatants of microglial cultures stimulated with Pam3CSK4 (*p* < 0.001 vs. Pam3CSK4). Similarly, lower IL-6 levels were detected in cells treated with Rif (*p* < 0.05 vs. Pam3CSK4) and RifQ (*p* < 0.01 vs. Pam3CSK4). As expected, α-TLR2 (2.5 μg/ml), an antagonistic antibody against TLR2 receptors [25,33], largely inhibited the induction of cytokine release (TNF-α and IL-6) by Pam3CSK4 (*p* < 0.05 vs. Pam3CSK4) (Figure 4B).

We also tested the impact of both antimicrobial drugs when microglial cells were activated by the purinergic P2X7 receptor agonist Bz-ATP. We observed that cytokine release was significantly reduced by Rif (*p* < 0.001 for TNF-α and *p* < 0.05 for IL-6 vs. bz-ATP) and was even more robustly reduced by RifQ (*p* < 0.001 vs. bz-ATP) (Figure 4C). We also observed that the synthetic antagonist of P2X7 receptors JNJ (20 μM), [34] reduced cytokine levels after bz-ATP treatments (*p* < 0.001 for TNF-α and *p* < 0.05 for IL-6 vs. bz-ATP).

Altogether, these results suggest that Rif and RifQ have the capacity to modulate inflammatory pathways activated through receptors that transduce the effects of αS_f_. However, RifQ consistently showed much stronger inhibitory effects than Rif on test inflammatory markers.

### 3.4. Inhibitory Effect of Rif and RifQ on αS_f_-Induced PI3K/AKT Activity

Previous studies have shown that the PI3K/AKT pathway may be essential for some of the inflammatory responses mediated by microglial cells [25,35,36,37,38]. On this basis, we studied the modulation of this signaling pathway in microglial cultures that were activated with αS_f_ and treated or not with Rif or RifQ. To this end, we assessed AKT phosphorylation by western immunoblotting of microglial cell lysates, using an antibody that recognizes phospho-Ser473-AKT.

**Figure 5:**
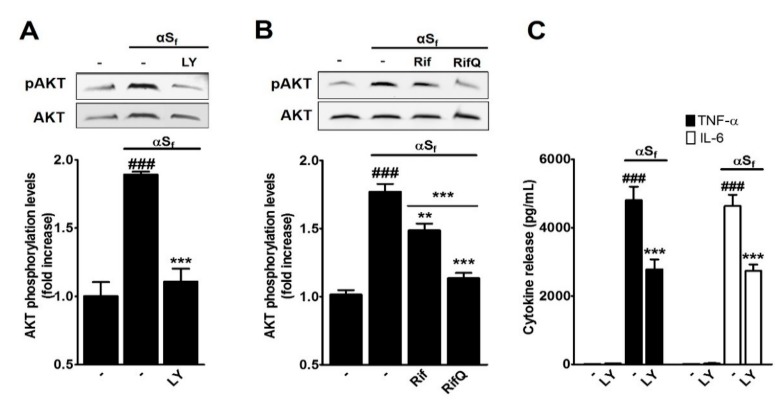
Rif and RifQ inhibit a PI3K-dependent mechanism. (**A**) Western blot analysis of protein kinase B (AKT) phosphorylation in control and αS_f_-treated cultures exposed or not to the PI3K-specific inhibitor LY (2.5 μM). (**B**) Western blot analysis of AKT phosphorylation in control and αS_f_-activated microglial cells treated or not with 100 μM of Rif or RifQ. A representative blot is included (top), and relative levels of pAKT/AKT are shown (bottom). (**C**) TNF-α (filled bars) and IL-6 (open bars) released by microglial cells pretreated or not with LY prior to stimulation with αS_f_. Values are shown as means ± SEM of four independent experiments performed in triplicate. Note: ^###^*p* < 0.001 vs. controls. **p < 0.01 vs. αS_f_ and ***p < 0.001 vs. αS_f_ or Rif *vs.* RifQ.

We observed a significant increase of AKT phosphorylation, 30 min after challenging microglial cultures with αS_f_. This increase was fully inhibited by LY, a selective inhibitor of PI3K, the kinase that phosphorylates AKT (Figure 5A). Both Rif and RifQ prevented AKT phosphorylation, although with different efficacies (Figure 5B). On average, the stimulation of microglial cells with αSf increased the phosphorylation of AKT by 1.77-fold (*p* < 0.001 vs. controls), while Rif and RifQ reduced this effect by 0.28- (*p* < 0.01 vs. αS_f_) and 0.63-fold (*p* < 0.001 vs. αS_f_), respectively. Statistical analysis revealed RifQ was more effective than its non-oxidized counterpart (*p* < 0.001) in inhibiting AKT activation triggered by αS_f_. In fact, RifQ appears as efficient as LY in this present setting. 

Note, however, that we observed only a 50% reduction in the release of TNF-α and IL-6 in αS_f_-treated cultures exposed to an optimal concentration of LY (*p* < 0.001 vs. αS_f_) (Figure 5C), whilst the efficacy of Rif and RifQ to inhibit the release of these two cytokines was generally much greater. This means that Rif and RifQ prevented the induction of cytokine release by inhibition of mechanisms that were presumably dependent and independent of PI3K signaling. Note that LY alone had no effect on basal cytokine release.

### 3.5. Rifampicin and Rifampicin Quinone Prevent Reactive Oxygen Species Production in Microglial Cells Activated by αS_f_

**Figure 6:**
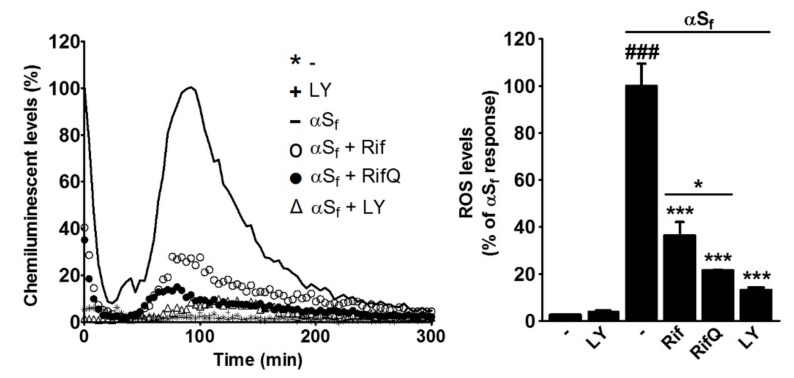
Rif and RifQ prevent reactive oxygen species production in microglial cells activated by αS_f._ Left panel shows a representative kinetic profile of superoxide formation as a percentage of the maximal response elicited by αS_f_. The light intensity was monitored continuously for 300 min. Experimental values in the right panel represent the means of the area under the curve in percentage of the maximal response elicited by αS_f_. Data are expressed as the mean ± SEM of four independent experiments performed in triplicate. Note: ^###^*p* < 0.001 vs. controls, *p < 0.05 Rif vs. RifQ, ***p < 0.001 vs. αS_f_.

To investigate potential effects of Rif and RifQ on ROS production, we used a luminol-based chemiluminescence assay to measure O_2_^•−^generation in αS_f_-stimulated microglial cells treated with or without antibiotics. Microglial cell cultures activated with αS_f_ showed a strong increase in O_2_^•−^production that peaked 90–100 min after initiation of the treatment and decreased progressively thereafter (Figure 6). This increase was strongly attenuated by Rif and RifQ treatment. Specifically, the ROS signal was estimated at 36.4% and 21.5% of the maximal response in αS_f_-treated cultures exposed to Rif or RifQ, respectively (*p* < 0.001 vs. αS_f_). There was again a significant difference in the efficacy of the two drugs (*p* < 0.05). Note that LY (2.5 µM) was also highly effective in inhibiting ROS production in the same setting. Indeed, ROS were estimated at 13.2% of the maximal response when αS_f_-treated cultures were concomitantly exposed to LY (*p* < 0.001 vs. αS_f_). LY alone had no effect on basal ROS production in this setting. Overall, our data reveal that Rif and RifQ reduced ROS production through inhibition of a PI3K/AKT-dependent mechanism.

### 3.6. Rifampicin Quinone Protects Cortical Neurons Against Death Caused by αS_f_-Induced Microglial Activation

**Figure 7:**
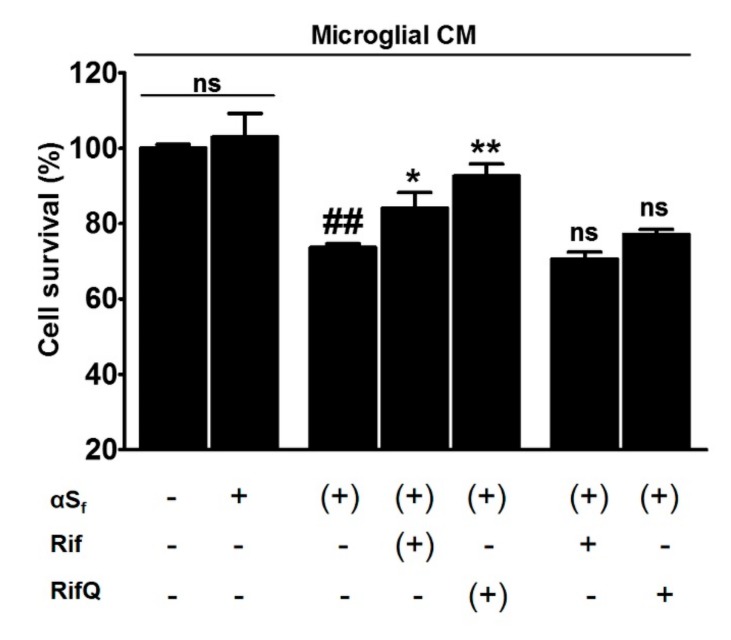
Rif and RifQ attenuate neuronal death caused by microglia activated by αS_f_. Cortical neurons were cultured for 48 h in the presence of microglial conditioned medium (CM) obtained as described in the text. (+): Indicates that the treatment was supplied initially to microglial cultures, and was thus provided indirectly to neuronal cultures through CM. The CCK-8 assay was used to evaluate neuronal viability. Data represent means of three different experiments performed in triplicate and error bars show SEM. Results are expressed as percentage of control values in cortical cultures receiving CM from control microglial cultures. Note: ^##^*p* < 0.01 vs. control CM; *p < 0.05, **p < 0.01 vs. αS_f_-treated CM; ns = not significantly different from αS_f_-treated CM.

**Figure 8:**
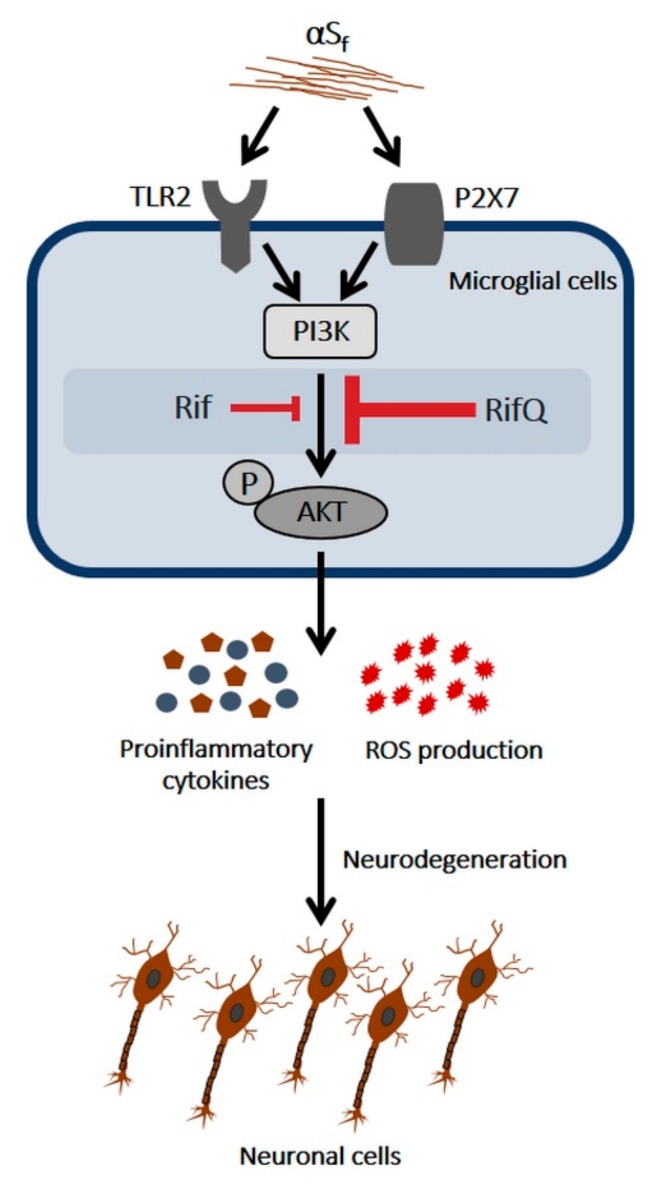
Simplified scheme describing how Rif and RifQ attenuate the inflammatory response of microglial cells exposed to αS_f_. Microglial TLR2 and P2X7 receptors recognize fibrils of αS, which results in AKT phosphorylation by PI3K. This leads to stimulation of cytokine release and to a burst of oxidative stress, presumably by activation of the reduced nicotinamide adenine dinucleotide phosphate oxidase enzyme. Both pro-inflammatory cytokines and ROS are potentially deleterious for neuronal cells. The suppressive effect of Rif and RifQ on cytokine release is probably due to inhibition of both PI3K and non-PI3K-dependent signaling events. The control of oxidative stress appears, however, essentially dependent on PI3K inhibition. The inhibitory effects that Rif and RifQ exert on activated microglia may provide indirect protection to neuronal cells. RifQ appears constantly more effective than Rif in reducing inflammatory-type reactions and in providing neuronal rescue.

It is currently unknown whether neuronal demise in synucleinopathies is due to a direct, cell-autonomous effect of αS species or whether it results from a non-cell autonomous effect due to a chronic exposure of neuronal cells to inflammatory mediators produced by activated immune cells. Thus, to test the neuroprotective potential of Rif and RifQ, we used a model system of neuronal cortical cultures, where treatments with αS_f_ were applied either directly to these cultures or indirectly through the use of microglial conditioned medium (CM). We observed that CM from αS_f_-stimulated microglial cultures caused a 26.4% loss of neuronal viability (Figure 7) as estimated with the CCK-8 test (*p* < 0.01 vs. controls). At variance, neurons were not affected when exposed directly to αS_f_ in the presence of CM from control microglial cultures, indicating that aggregated forms of αS were toxic only though their stimulatory effect on microglia. Interestingly, the medium conditioned by microglial cultures activated by αS_f_ was less neurotoxic if the conditioning step was performed in the presence of Rif or RifQ (Figure 7). Precisely, neuronal viability was decreased by only 16% (*p* < 0.05 vs. αS_f_-treated CM) and 7% (*p* < 0.01 vs. αS_f_-treated CM) in these two conditions, respectively. This means that RifQ was also more efficient than Rif in inhibiting microglial-dependent neurotoxicity. Note that neither Rif nor RifQ afforded neuroprotection when applied to cortical cultures receiving CM from αS_f_-treated microglial cells. All experimental results are summarized in Figure 8.

## 4. Discussion

The results presented herein show for the first time that Rif and its oxidized derivative RifQ prevent the activation of primary microglial cells induced by αS_f_, a relevant inflammogen in the context of Parkinson’s disease. In our experimental paradigm, Rif and RifQ treatment inhibited key markers of inflammation, with RifQ being consistently more efficacious than Rif. We observed that the anti-inflammatory action of these two drugs required inhibition of PI3K-dependent and independent signaling events. Importantly, we demonstrated that RifQ had the potential to prevent neurotoxic effects elicited by αS_f_-activated microglial cells.

### 4.1. Fibrillary Aggregates of αS are the Most Inflammogenic Forms of αS for Microglial Cells

The aggregation of the synaptic protein αS represents one of the essential pathological events in PD. It has been suggested that pathological forms of αS that spread in the brain parenchyma [39,40] could contribute to disease progression by inducing inflammatory-type reactions mediated by microglial cells [41]. In the context of this study, we tested the inflammatory potential of three different forms (i.e., monomeric, oligomeric, and fibrillary) of the protein, using a model system of pure microglial cell cultures. Of note, endotoxins potentially present as artefactual contaminants in recombinant human αS were removed using a high capacity endotoxin removal resin.

When using the release of TNF-α and IL-6 as inflammation markers, we found that αS_f_ were the most inflammogenic forms of the protein. This is consistent with reports showing that the inflammatory potential of αS depends on the aggregation state of the protein [41,42]. Present results are also in line with data from Gustot et al. (2015) [43], who previously reported that αS fibrils were accountable for the activation of a THP-1 monocyte cell line and the release of IL-1β through the activation of TLR2 and NLRP3 [43]. Additionally, Hoffmann et al. (2016) [42] showed that αS fibrils were more effective in increasing the production and secretion of pro-inflammatory cytokines in microglial BV-2 cell cultures compared to monomeric and oligomeric species [42]. Moreover, Chavarria et al. (2018) [44] recently reported that rat astrocytes incubated with αS induced hippocampal neuronal death in a co-culture experimental setting and that neurotoxicity was particularly enhanced by exposure to fibrillar αS [44]. These studies reinforce the idea that αS aggregated species are principally responsible for the activation of immune system cells. Therefore, in all subsequent studies we used aggregated forms of αS to test the anti-inflammatory potential of Rif and RifQ.

### 4.2. RifQ is More Efficient than Rif in Reducing αS_f_-Induced Microglial Cell Activation

We then wanted to compare the anti-inflammatory effects of Rif and RifQ in αS_f_-activated microglial cultures. It has been reported previously that Rif suppresses the activation of BV-2 microglial cells induced by the inflammogen LPS and by the mitochondrial toxin rotenone [18,20]. In concordance with these results, we show that Rif and also RifQ decreased the production of pro-inflammatory cytokines in microglial cells stimulated by αS_f_. This of interest as pro-inflammatory cytokines have been identified as key mediators of neuronal damage and demise in several models of PD [44,45,46]. Note that we observed that this anti-inflammatory effect was significantly greater in cultures treated with RifQ. The better efficacy of RifQ was also observed when measuring IBA-1 expression [29,30] and ROS production as microglial activation markers. Such differences in activity between the two compounds suggest indirectly that Rif did not undergo significant autoxidation into RifQ in our conditions. The observation that Rif and RifQ not only reduced the release of pro-inflammatory cytokines but also the production of ROS in microglial cells activated by αS_f_ is of interest. Indeed, ROS can promote neuronal death and prevention of oxidative stress-mediated insults could possibly offer an effective therapeutic approach to mitigate the progression of neurodegenerative diseases, such as PD.

### 4.3. Rif and RifQ Inhibit an Activation Process Mediated by TLR2 and P2X7 Receptors

The ability of amyloid-like structures to bind cell membrane components probably explains why αS_f_ have been found to interact with transmembrane signal transducing receptors [47]. We previously found [25] that glutamate release induced by αS_f_ was largely reduced in the presence of an antagonistic antibody against TLR2, a TLR subtype that is probably involved in PD-associated brain inflammation [48,49,50]. Glutamate release induced by αS_f_ was also curtailed by JNJ, a synthetic antagonist of the purinergic P2X7 receptor, reported by others as a putative receptor for αS [35]. We also found here that both receptors were involved in the proinflammatory effects of αS_f_. With this in mind, we tested the efficacy of Rif and RifQ against specific agonists of TLR2 (Pam3CSK4) and P2X7 receptors (bz-ATP) and found that Rif and RifQ inhibited cytokine release induced through activation of each receptor. Note that in this context, RifQ was again more efficacious than Rif in reducing microglial inflammatory responses.

These data also suggest indirectly that Rif and RifQ controlled the effects of αS_f_ by presumably interfering with the signaling pathways activated by TLR2 and P2X7 receptors rather than by reducing the inflammogenic potential of fibrils by possibly forming complexes with them. However, the possibility that Rif and RifQ could bind directly to TLR2 and P2X7 receptors, thereby preventing their stimulation by αS_f_, is not totally excluded. We should finally mention that we did not test the effects of Rif and RifQ against other membrane receptors that might also participate in αS_f_-microglial activation. In that respect, β1-integrins and TLR4 have been reported to also convey some inflammatory effects of αS species [51,52,53].

### 4.4. Rifampicin and Rifampicin Quinone act as Inhibitors of PI3K/AKT-Dependent Signaling

To further understand how Rif and RifQ antagonized the effects of αS_f_, we treated microglial cells with αS_f_ and estimated expression levels of phospho(activated)-AKT. We showed that αS_f_ induced the phosphorylation of AKT, which is consistent with the implication of this protein kinase in signaling events mediated by TLR2 and P2X7 receptors [35,54], i.e., putative receptors for αS_f_. Interestingly, treatments with Rif and RifQ were effective in reducing AKT activation, with the efficacy of RifQ being equivalent to that of the PI3K inhibitor LY and more pronounced than that of Rif. This suggested that the inhibition of PI3K/AKT signaling accounted for the anti-inflammatory effects of Rif and RifQ. It should be noted, however, that an optimal concentration of LY was proportionally more efficacious in lowering oxidative stress than cytokine release, suggesting that non-PI3K-dependent signaling events also intervened in the inhibitory effects that Rif and RifQ exert on cytokine release.

### 4.5. Rifampicin Quinone Provides Neuroprotection through its Anti-Inflammatory Activity

Despite the fact that midbrain dopamine neurons are considered to be the main target of the disease process in PD [55], there are other brain neuronal populations also affected in this disorder, in particular in the cerebral cortex [56,57]. In that regard, there is an increasing amount of evidence for cortical involvement in early and prodromal stages of PD [58], making primary cortical neurons in culture, a valuable tool for studying neuroprotection by Rif and RifQ [59].

Previous studies have shown that Rif was able to improve neuronal survival by inhibition of inflammatory processes induced by LPS-activated microglial cells [17,18]. Here, we wanted to determine whether inflammation products released by microglial cells exposed to αS_f_ could induce neuronal death and whether RifQ and Rif could prevent these effects. We demonstrated that CM from αS_f_-activated microglia promoted neuronal cell death in cortical cultures, whereas CM from non-activated (control) microglia failed to do so. This is in agreement with other studies showing that microglial cells activated with αS_f_ or other αS species can induce neuronal damage in other experimental settings [35,60,61].

We found that the medium that was conditioned by microglial cells exposed to αS_f_ in the presence of RifQ caused less neuronal damage than the medium conditioned by microglial cells solely exposed to αS_f_. Rif was more modestly protective in the same paradigm. When RifQ and also Rif were added directly to cortical cultures receiving CM from αS_f_-treated microglia, no neuronal rescue was observed, suggesting that neuroprotection was indirect and resulted from an anti-inflammatory effect on microglia. The fact that RifQ had better efficacy than Rif in reducing inflammatory responses in αS_f_-treated microglial cell cultures explains most probably why neuroprotection was preponderant with RifQ in the present setting.

Present results reinforce the idea that inflammatory microglial cells release molecules that are potentially toxic to neurons and that compounds such as RifQ may prevent neuronal damage through their suppressive effect on microglia. Note, however, that in a PC12 culture model, Rif was reported to reduce the overexpression of αS provoked by exposure to the neurotoxicant MPP^+^ [62,63], which suggests that in a pathological context, Rif or RifQ may also protect neuronal cells by reducing the αS load.

Overall, present data demonstrate that RifQ exerts more potent anti-inflammatory effects than its parent compound Rif, in a setting where microglial cells are activated by fibrillary aggregates of αS, a potential trigger for PD-induced neurodegeneration. The immunosuppressive effect of RifQ on αS_f_- activated microglial cells appeared to be sufficient for providing protection against neuronal cell death. Thus, data with RifQ appear promising enough to justify further studies to confirm the potential of this compound as an anti-parkinsionian drug. In particular, these studies should demonstrate whether neuroprotection remains achievable after delayed intervention with RifQ in relevant PD models.
